# Longitudinal evaluation of a knowledge translation role in occupational therapy

**DOI:** 10.1186/s12913-019-3971-y

**Published:** 2019-03-12

**Authors:** Danielle Hitch, Kate Lhuede, Lindsay Vernon, Genevieve Pepin, Karen Stagnitti

**Affiliations:** 1 Deakin University, Waterfront Campus, 1 Gheringhap Street, Geelong, Victoria 3217 Australia; 2 North Western Mental Health, Waratah Clinic, 641 Mt. Alexander Road, Moonee Ponds, Victoria 3039 Australia; 3 North Western Mental Health, North West Area Mental Health Service, 130 Bell Street, Coburg, Victoria 3058 Australia

**Keywords:** Occupational therapy, Knowledge translation, Evidence based practice, Knowledge brokerage, Allied health, Research

## Abstract

**Background:**

In 2014, a large metropolitan mental health service in Australia developed a senior role (Lead Research Occupational Therapist) to address an identified need for greater research and knowledge translation, and associated capacity building. The aim of this study was to evaluate the impact, in the first 2.5 years, of this role across a range of variables.

**Methods:**

Multiple methods were used to gather a comprehensive range of data. Workforce surveys were completed both online and in hard copy in early 2014 (*n* = 42) and late 2016 (*n* = 44). Research key performance indicators (academic, research production and cultural) were also identified for measurement over time. The data from these surveys were analysed using descriptive and inductive analysis, and also with social network analysis.

**Results:**

This role has demonstrated positive outcomes across a range of variables. There was a medium effect on the quantity of participation in quality assurance and knowledge translation activities by the workforce. Most knowledge translation behaviours were occurring regularly, although several were absent. An improving trend in attitudes towards evidence-based practice was recorded, and perceptions of the knowledge translation role were generally positive. The Lead Research Occupational Therapist moved from the periphery to the centre of the evidence based practice social network. Improved awareness of other clinicians deploying evidence based practice was observed, and the frequency of interaction between clinicians increased. The role has met all key performance indicators, across the academic, research production and cultural domains.

**Conclusions:**

The shift in focus of this role from research to knowledge translation has produced tangible outcomes for the occupational therapy workforce. These achievements have had a positive impact on the sustainability of the role, which will be continued for at least another two years. An ongoing challenge is to directly measure the impact of this role on outcomes for people with mental illness and their carers.

## Background

Knowledge translation and research capacity within allied health has been increasingly recognised as a crucial aspect of evidence based practice. The classic definition of evidence based practice focused on “making decisions about the care of the individual patient” [1], and did not necessarily account for the interpersonal, contextual and group or service wide application of evidence to practice. Curriculum around evidence based practice in allied health has focused almost exclusively on the initial phases of the evidence based practice process – ask, acquire and appraise – but has neglected the final, arguably most important steps – applying and analysing the impact of evidence based practice [2]. The application of evidence into practice has therefore remained the ‘missing link’.

Knowledge translation related to health has been described as “a dynamic and iterative process that includes the synthesis, dissemination, exchange and ethically sound application of knowledge to improve health, provide more effective health services and products, and strengthen the health care system” [3]. To enable this application of knowledge to practice, the allied health workforce must have sufficient capacity to access, critique, adapt and embed evidence into their daily practice. In addition, allied health clinicians may also need the capacity to generate quality research, should there be insufficient evidence available to address their clinical needs.

The allied health research leadership position described here was originally titled the Occupational Therapy Academic Project Officer. Over time, the position title evolved to become Lead Research Occupational Therapist after feedback that the original title implied a short term, small scale remit. The aim of the position is to provide leadership and vision around embedding research into occupational therapy practice at the service, with the key deliverables including completion and publication of research projects, formulation of a strategic plan to build research capacity and culture, formulation of a database of research activity at the service and development of documentation and resources to support the ongoing sustainability of the position. The key knowledge and experience required for the position included relevant qualifications in occupational therapy (preferably, but not necessarily, at PhD level), sound knowledge and understanding of occupational therapy clinical practices in public mental health, the ability to manage complex projects and a commitment to consumer focus and continuum of care.

This position focused on building research capacity (in regards to the consumption and generation of research) as a complementary and simultaneous process to building capacity for knowledge translation (through the adaptation of evidence to local contexts). All research sourced and/or generated within the service over the course of this study was explicitly framed within the needs of the local organisation and community, and considered in regards to its potential impact on occupational therapy practice at that service.

The study reported here occurred within the Australian healthcare system, where several recent studies have investigated measures aimed at increasing allied health research capacity. Australian allied health clinicians have indicated they are motivated to participate in research by intrinsic factors, such as a personal interest, while team motivators were related to delivering the best service possible and achieving the best outcomes for patients [4]. Occupational therapists in particular have reported they enjoyed working in a department with a visible research and evidence based culture, which led to feelings of pride and confidence, but also pressure [5]. Time pressures during work hours and a lack of confidence in skills were also flagged as key barriers, which has been a consistent finding across time and disciplines [6–10].

The role of leadership in supporting research capacity has also been explored, with the development of local models to guide research and application to practice found to be supportive [11]. Other examples of multi modal research capacity building initiatives at an organisational level have also been recommended or described, with positive outcomes reported for academic outputs [12] and proposed for clinical practice [13, 14].

The creation of leadership positions specifically targeted at research and evidence based practice is also an emerging strategy in allied health. A systematic review [15] has identified the main focus of these positions were to provide academic support to clinicians throughout the research cycle, develop academic research and provide service level / organisational support. The impact of these positions reported in the literature included traditional academic outcomes (writing and dissemination, acquisition of funding, research performance outcomes, data collection and analysis, development of research skills at the individual, team and organisational levels, increased research activity), and cultural outcomes (improved research culture, improve profile of allied health and attitudes towards research) [14–18] .

However, only two of these studies identified knowledge translation outcomes, citing specific examples of clinical and service changes which resulted directly from the research undertaken. These examples were reported anecdotally, and were yet to be comprehensively evaluated at the time of these studies. The focus on research capacity in allied health highlights a clear gap in currently knowledge, which targets the generation of knowledge rather than its application into clinical practice.

Only one published study to date has sought to undertake a longitudinal evaluation of the impact of research positions in allied health [16], which was conducted over the duration of a year and focused on a particular research study. While several studies have commented on the assumed positive impacts of research positions (largely in relation to research outcomes), none have sought to measure them objectively and all evidence to date has originated from physical health settings. This study therefore makes a unique contribution to the growing evidence base around knowledge translation in allied health. It is the first to use multiple methods to evaluate the impact of a position similar to those previously investigated, although with more explicit focus on knowledge translation. It also describes impact in a mental health service, which has a substantially different service culture than those found in physical health.

### Aim

The aim of this study was to evaluate the impact of a leadership position for knowledge translation in occupational therapy in the first 2.5 years. To achieve this aim, the study sought to describe:

1. changes in the workforces’ participation in quality assurance, research and knowledge translation activities (such as changes to practice, revisions of documentation, dissemination in multiple formats etc).
2. changes in the attitudes of the workforce towards evidence based practice
3. workforce perceptions of this knowledge translation role
4. changes in the social network in this service around evidence based practice
5. outcomes achieved by this role against key performance indicators

## Methods

### Setting

The setting of this study was a public mental health service for people living in a major Australian city. This service delivers a comprehensive range of specialist, community and hospital-based mental health services for youth, adult and older people who are experiencing, or are at risk of developing a serious mental illness. The service comprises 6 programs, located in 32 separate sites across the catchment area. It also includes tertiary specialist services for neuropsychiatry and eating disorders. The current occupational therapy workforce numbers 90 individuals, or the equivalent of 70 full time positions. An organisational diagram to illustrate structure of the occupational therapy service at this service has been included as supplementary information for context.

### Data collection

This study used multiple methods to measure the impact of the occupational therapy leadership role in research and knowledge translation, and received ethics approval from the service’s Office of Research (QA2014029). Data was collected via workforce surveys in March–April 2014 and November–December 2016. Workforce surveys had been undertaken previously at the service around recruitment and retention, workplace culture and safety issues [19]. However, the two surveys reported here focused specifically on outcomes related to research, evidence based practice and knowledge translation.

Participants were recruited via an email sent by local Chief Occupational Therapists in each of the 6 programs. The email included a link for online completion of the survey using an institutional Survey Monkey account. Paper copies of the survey were also distributed at monthly discipline meetings, to provide an alternative method of participation. These paper copies were returned to the principal researcher, and entered into the Survey Monkey account, before being securely shredded. A follow up email was sent after two weeks, to prompt further participation and thank those who had returned their survey. The survey remained open for responses for a further two weeks (four weeks in total).

The first page of the survey provided a plain language statement, which highlighted the voluntary nature of participation along with the context of the study. If the participant provided consent, they were taken through to the rest of the questions. The survey had four sections, and began with open and closed demographic questions (About You). One of these questions asked participants if they had participated in a quality assurance or research activity in the previous years, focusing on participation in projects that had been formally reviewed by the service’s Office of Research. Participants were then asked to complete the Evidence-Based Practice Attitude Scale (EBPAS-15) [20]. The third part of the survey collected data about the participant’s evidence based practice social network, with questions asking for identification of key contacts and frequency of contact. Finally participants were posed open and closed questions about the Lead Research Occupational therapist role, and what they wanted to see in the next iteration of the occupational therapy services’ research strategy. A series of 18 characteristics of the role were surveyed, derived from Wenke and Mickan’s systematic review [15], with participants asked to rate the incumbents performance on a scale of 0–5 (0 = poor and 5 = excellent). This section of the survey was only administered in 2016, as the role had not been established long enough in 2014 to gain meaningful valid data.

### Outcome measures

The majority of the survey consisted of bespoke questions, but two standardised outcome measures were included – the Evidence Based Practice Attitude Scale (EBPAS-15) [20] and the Evidence Based Practice Implementation Scale [21]. The EBPAS-15 measures mental health clinician attitude towards evidence based practices in healthcare, and was administered in both 2014 and 2016. The scale uses a Likert format, with 15 questions related to four subscales – appeal (4 questions), requirements (3 question), openness (4 questions) and divergence (4 questions) [20]. The 5-point scale for each statement is rated from 1 (not at all) to 5 (very great extent) with higher scores indicating more positive attitudes. This scale has confirmed internal consistency reliability.

The psychometric properties of the EBPAS-15 have been established in several studies [22–24]. Moderate to excellent reliability is reported for the subscales and total scale score in a sample of mental health service providers [25], and was confirmed in a further study with a more geographically diverse sample [22]. The content validity of this tool was established during its development from a literature review and consultation with both mental health service providers and researchers [20]. Good construct and convergent validity has also been reported in relation to measures of mental health organisational structure [20], and organisational culture and climate [23, 26].

The Evidence Based Practice Implementation Scale [21] measures changes in practice around research and knowledge translation, and was only administered in 2016 for benchmarking [21]. The original scale has 18 items, but only the 10 knowledge translation items were utilised in this study. Research into the psychometric properties of this scale has provided some evidence for its reliability and validity [21]. In a heterogeneous sample of nurses, the scale was found to have excellent internal consistency; good construct validity and very good criterion validity [21]. Versions of the scale translated into Slovak and Czech also demonstrated good to excellent internal consistency and established cross cultural validity [27].

### Data analysis

All data analysis for this study in based on description of the sample characteristics, and was completed using SPSS Version 25 software and socnetv. Means and frequencies have been used to describe the workforces participation in quality assurance and knowledge translation activities, attitudes towards evidence based practice, perceptions of the knowledge translation role, changes in the social network and outcomes for key performance indicators.

Distribution was assessed using Q-Q plots and found to be non-parametric. Therefore, Mann Whitney U Tests and Chi Square Tests were used to analyse any differences between the samples in 2014 and 2016. Due to the data not being normally distributed, Cliff’s Delta was used to analyse changes in quality assurance and knowledge translation activities. Cliffs Delta is suitable for non-parametric datasets, as it makes no assumption about the shape or spread of the data distribution [28]. This statistic cannot be calculated in SPSS currently, and so calculations were made using a specially designed Excel macro which also calculated the equivalent effect size using Cohen’s d [29]. A small effect for Cohen’s d is a result between 0 and 0.2, a medium effect is 0.3 to 0.6 and a high effect is 0.7 and above [30].

Social network analysis has emerged from social science in recent years, and aims to consider social relationships in terms of network theory [31]. In particular, it focuses on the relationships between groups of individuals and the resources to which membership of these groups facilitate access [32]. It is a method which highlights the relationship between individual behaviour and systemic change [33]. While social network analysis has been used in Australian mental health services before [34], this study is one of the first to apply it to knowledge translation in health. De-identified data from the survey was entered into Social Network Visualiser (socnetv). This program converted the data into an adjacency matrix and network graph, showing connections that were analysed as a complete network (i.e. including contacts both in and outside of occupational therapy). This is a binary directional network, showing the direction of communication between nodes, and the cognitive social structure within the service associated with evidence based practice [33]. This network is only relevant to communication around evidence based practice and knowledge translation, and the representation of this service may be significantly different that that found in other services for the same topic.

The following metrics were analysed descriptively – nodes, arcs, density, distance, diameter, average clustering coefficient, and centrality. Nodes are represented as circles on the network graph and represent individual clinicians [32]. Arcs are the lines that connect each node, which represent the direction of each relationship. Density is calculated by the total number of arcs divided by the total possible number of arcs, and expresses how aware clinicians are of each other [32]. Distance measures the minimum number of arcs between two clinicians, while diameter refers to the longest of the possible paths between clinicians [35]. The average clustering coefficient indicates the degree to which clusters of clinicians are evident within the network, while centrality illustrates how often clinicians interact with others in the network [36].

Finally, the outcomes of this position were reported against a set of key performance indicators established at its commencement. These indicators were developed from both an academic perspective (reflecting the traditional metrics of this sector) and a knowledge translation perspective. The indicators have evolved over time, and are now formalised in the annual occupational therapy research strategy at this service. This strategy is aligned with the service’s organisational mission and its research committee multidisciplinary research plan. These outcomes include a further year of data collection, and reflect the impact of the position over 3.5 years.

### Participants

A total of 86 responses were received from occupational therapists participating in this study; 42 in 2014 and 44 in 2016. Some of the participants participated in both surveys, but due to workforce turnover, some only completed one survey. It was not possible to track individual participant answers due to confidentiality requirements. Participants came from all 6 programs, and the majority were working in the community. Participants were also drawn from a range of experience levels, with some working in management and generic positions also participating. The majority of participants had Bachelor qualifying degrees, and a substantial number were studying for higher degrees (*n* = 21, 50% in the first sample *n* = 27, 61% in the second sample). Chi square and Mann Whitney U analysis resulted in no significant differences between the two samples on any of these variables, which are illustrated below in Table 1. Please note that not all participants answered all demographic questions, and some participants worked across several settings. As shown in the Table, there were relatively few demographic differences between the samples that responded to the two surveys, although the second sample did have more variation in regards to years of service as both an occupational therapist and in mental health.

**Table 1:** Demographic Profile of Samples

	2014 (*n* = 42)	2016 (*n* = 44)
Area of Practice	Aged = 8 Youth = 8 Adult = 28	Aged = 8 Youth = 8 Adult = 22
Setting	Inpatient = 9 Community = 34	Inpatient = 6 Community = 31
Grade	1 = 7 2 = 19 3 = 14 4 = 0 Other = 4	1 = 9 2 = 12 3 = 11 4 = 4 Other =2
Education	Bachelors 37 Obtained Graduate Certificate 3 Obtained Graduate Diploma 6 Obtained Masters 7 Obtained, 5 Working towards Professional Doctorate N/A PhD N/A	Bachelors 31 Obtained Graduate Certificate 5 Obtained Graduate Diploma 9 Obtained Masters 10 Obtained Professional Doctorate I Obtained PhD I Obtained, 1 Working Towards
Average Service as an Occupational Therapist	7.98 years SD = 6.14 Range 0.5 years – 25 years	11.39 years SD = 9.34 Range 1 year - 40 years
Average Service in Mental Health	7.50 years SD = 5.72 Range = 0.5 years – 24 years	10.35 years SD = 7.77 Range = 0.5 years – 30 years

## Results

### Participation in quality assurance and knowledge translation activities

The average number of formal quality assurance and research activities undertaken by the workforce was 0.74 in 2014 (SD = 1.18, Range = 0–4), and 1.60 in 2016 (SD = 1.64, Range = 0–5). A medium effect size was identified with Cliff’s Delta = 0.44 (95% CI [0.22, 0.62]). A greater proportion of participants self-identified as research generators (26 to 34%) and leaders (7 to 11%) in the second sample, but these changes were not statistically significant.

As shown in Table 2, at the time of the second survey participants engaged in the majority of knowledge translation activities surveyed using the Evidence Based Implementation Scale 1–3 times in the previous two months. However, no participants evaluated the impact of evidence based practice on patient, clinicians and systems outcomes formally, or shared the outcomes of quality assurance and research with patients and carers.

**Table 2:** Frequency of Evidence Based Practice Behaviours in past two months

	0	1–3	4–7	8+
Shared the outcomes of quality assurance activities / research with discipline colleagues verbally (*n* = 32)	*n* = 5, 15.62%	*n* = 22, 68.75%	*n* = 5, 15.62%	*n* = 0
Shared the outcomes of quality assurance activities / research in the form of a report or presentation to discipline colleagues (*n* = 32)	*n* = 14, 43.75%	*n* = 17, 53.12%	*n* = 1, 3.12%	n = 0
Shared the outcomes of quality assurance activities / research with colleagues from a different discipline (*n* = 31)	*n* = 15, 48.39%	*n* = 16, 51.61%	*n* = 0	n = 0
Changed your clinical practice as a result of quality assurance activities / research (*n* = 31)	*n* = 9, 29.03%	*n* = 22, 70.97%	*n* = 0	n = 0
Evaluated the impact of evidence based practice on patient outcomes formally (i.e. through quality assurance activities or study) (*n* = 33)	*n* = 27, 81.81%	*n* = 6, 18.19%	*n* = 0	*n* = 0
Evaluated the impact of evidence based practice on patient outcomes informally (i.e. through observation or discussion) (*n* = 28)	*n* = 10, 35.71%	*n* = 14, 50.00%	*n* = 4, 14.29%	*n* = 0
Evaluated the impact of evidence based practice on clinician or system outcomes formally (though quality assurance activities or study) (*n* = 33)	*n* = 25, 75.75%	*n* = 8, 24.25%	*n* = 0	*n* = 0
Evaluated the impact of evidence based practice on clinician or system outcomes informally (though observation or discussion) (*n* = 32)	*n* = 13, 40.62%	*n* = 17, 53.12%	*n* = 2, 6.25%	*n* = 0
Promoted the utilisation of outcomes from quality assurance activities / research to colleagues (*n* = 33)	*n* = 12, 36.36%	*n* = 19, 57.57%	*n* = 2, 6.07%	*n* = 0
Shared the outcomes from quality assurance activities / research with patients or carers (*n* = 32)	*n* = 23, 71.87%	*n* = 7, 21.87%	*n* = 2, 6.25%	*n* = 0

### Attitudes towards evidence based practice

There were no significant differences between the EBPAS-15 scores over time. However, all subscales scores changed in a positive direction over time (as illustrated in Table 3), as indicated by either increased or decreased mean scores.

**Table 3:** Subscale and overall mean scores and confidence intervals (95%) on the EBPAS-15

	2014 (*n* = 41)	2016 (*n* = 31)	Significance
Appeal	2.95 (CI 2.70, 3.20)	3.07 (CI 2.83, 3.31)	*U* = 563.00, *p* = 0.40
Requirements	2.56 (CI 2.27, 2.85)	2.62 (CI 2.40, 2.84)	*U* = 626.50, *p* = 0.92
Openness	2.48 (CI 2.26, 2.70)	2.50 (CI 2.27, 2.73)	*U* = 586.00, *p* = 0.57
Divergence	0.85 (CI 0.57, 1.13)	0.65 (CI 0.42, 0.88)	*U* = 503.50, *p* = 0.13
TOTAL	2.83 (CI 2.57, 3.09)	2.91 (CI 2.66, 3.16)	*U* = 630.50, *p* = 0.95

Note: *EBPAS-15* = Evidence Based Practice Scale 15, CI = confidence interval

### Perceptions of knowledge translation role

As shown in Table 4, the average ratings provided by the workforce after it had been established for 2.5 years indicated a positive perception of the Lead Research Occupational Therapist role.

**Table 4:** Perceptions of characteristics of the knowledge translation role (*n* = 35)

	Average (Scale 0–5)	SD
Discipline specific	4.13	0.78
Useful	4.07	1.05
Up to date	4.03	1.00
Supportive	3.90	1.12
Responsive	3.83	1.02
Promoting research	3.80	1.03
Informative	3.73	0.94
Communication	3.72	1.03
Innovative	3.70	1.21
Leadership	3.70	1.06
Links to academia	3.67	1.09
Capacity building	3.67	1.18
Consultation	3.60	1.00
Accessible	3.57	1.14
Culture building	3.57	1.04
Multidisciplinary	3.53	0.90
Collaboration	3.50	1.11
Facilitation	3.43	1.01

Note: *SD* = standard deviation

The workforce generally requested contact with the Lead Research Occupational Therapist on an occasional or monthly basis, and these preferences did not change significantly from the roles initial months in 2014. However, there was a significant shift in the method of contact requested over time. A Chi Square analysis confirmed that the number of occupational therapists preferring online contact rose significantly between 2014 and 2016 - *X*^2^ (130.00, *N* = 131) = 8.19, *p* = .04. In the first 2.5 years of the position, preferences for face-to-face, telephone and meeting contact decreased, although not to a statistically significant degree.

### Social network analysis

**Fig. 1:**
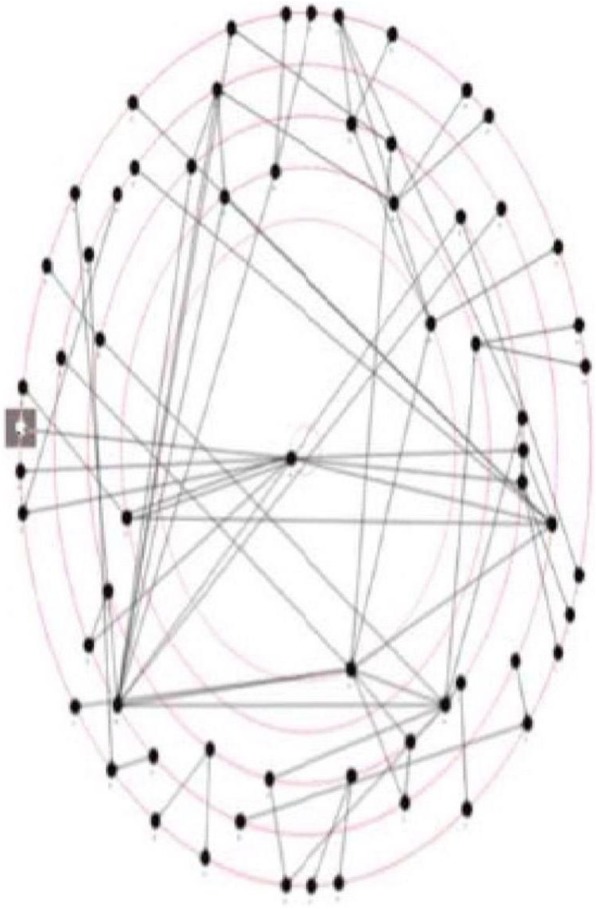
Social network for evidence based practice in 2014

**Fig. 2:**
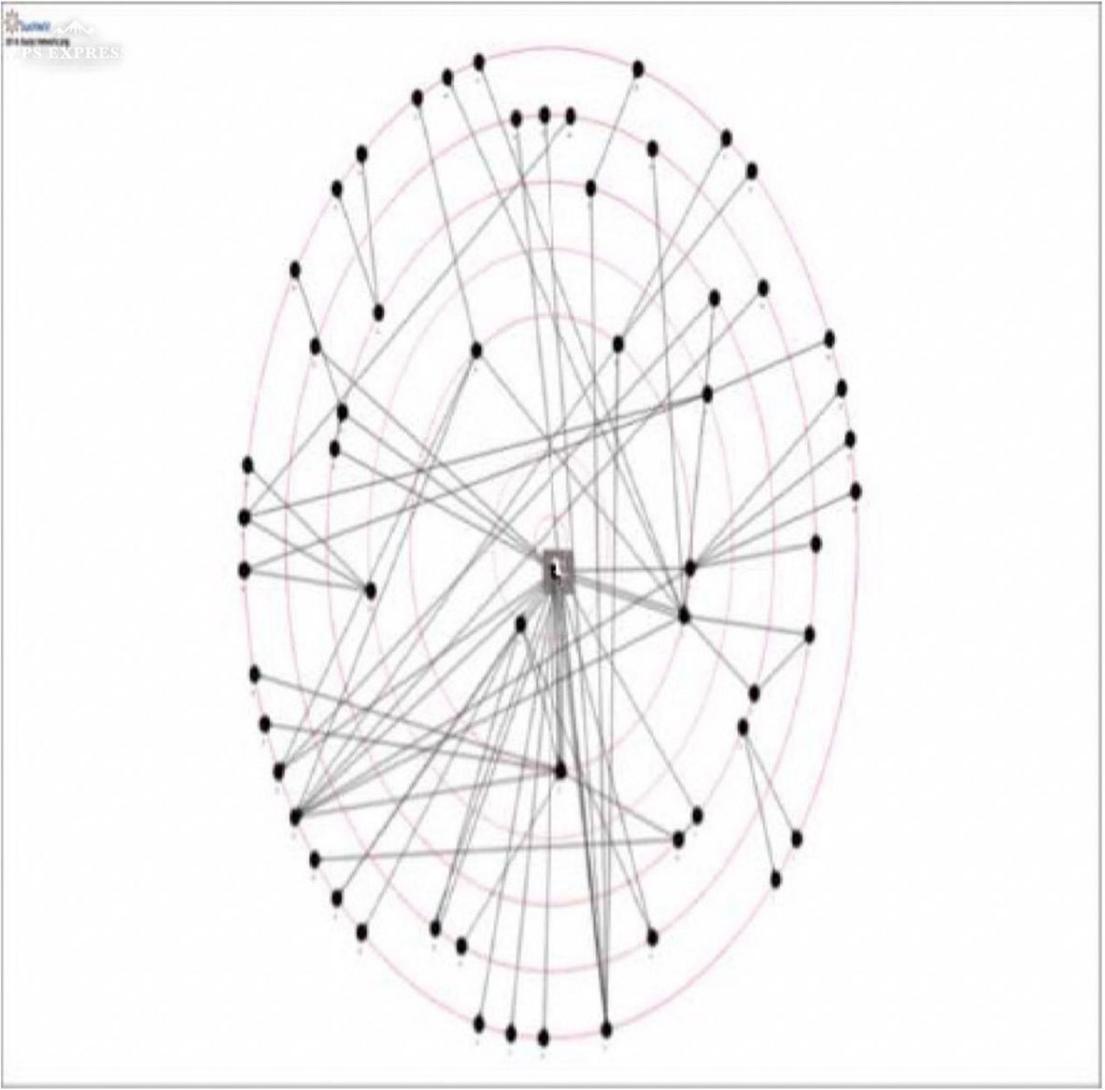
Social network for evidence based practice in 2016

As shown in Figs. 1 and 2, the social network around evidence based practice showed more connections between clinicians, and less bottlenecks (where a single clinicians is the only point of contact between areas of the network). The Lead Research Occupational Therapist position is highlighted in grey, and has moved from the periphery to the centre of the network over time.

As shown in Table 5 below, less nodes (i.e. evidence based practice contacts) were identified in 2016, but a comparable number of arcs. This reflected an increase in the links between, and awareness of clinicians within the network. A considerable amount of homophily was demonstrated by the clinicians within the inner circle of the network, who were all Chief Occupational Therapists and therefore similar to each other [33]. The diameter of the network also increased, along with the degree of clustering of clinicians. The metrics also indicate a modest increase in the frequency of interaction between clinicians in the network.

**Table 5:** Social Network metrics over time

Metric	2014	2016
Nodes	65	59
Arcs	74	75
Density	0.01779	0.02192
Distance	1.76364	2.66573
Diameter	5	6
Average clustering coefficient	0	0.0056497
Centrality	0.0017014	0.0017323

### Outcomes against key performance indicators

As shown in Table 6, all key performance indicators have been met over the first 3.5 years of the Lead Research Occupational Therapist position. The 28 active research projects currently underway involve the active participation of 41 individual clinicians, which represents 46% of the total service workforce.

**Table 6:** Outcomes for Key Performance Indicators

Domain	Indicator	Outcome
Academic	Undertake and develop specific research projects / streams in collaboration with clinicians	10 clinician led articles published or accepted for publication [52–60], with a further 5 currently under peer view 100% conversion rate to publications (either published or in development) for research projects to date 17 conference presentations at local, state and international conferences $150,000 AUD in grant and fellowship funding
	Supervision of higher degree by research students	Three students successfully completed (honours and masters) and one student current enrolled (honours).
Research Production	Undertake and develop specific research projects / streams in collaboration with clinicians	28 active research projects currently underway – 7 in data collection phase, 5 in the data analysis phase and 16 in the dissemination phase. Projects in all program areas – youth, adult and aged mental health.
	Individual and group mentorship of clinicians and other stakeholders at all stages of the research and knowledge translation process	1:1 mentorship of a named clinician for each current project
Research Culture	Supporting the translation of research and knowledge into practice, by embedding evidence into policies and practices across the organisation	Five or more examples of knowledge translation identified each year. E.g. changes to documentation practices, implementation of guidelines, increased secondary consultations
	Undertake leadership roles within networks and governance structures, and exert positive influence through these roles	Occupational therapy representation in all Research Committee, Consumer and Carer Advisory, Discipline Lead, Executive and local service meetings.
	Adoption of co-production as a guiding principle for the occupational therapy research program	Process for co-production currently being developed collaboratively with Consumer and Carer Advisory Group
	Establishment and maintenance of strategic collaborations and networks at multiple levels (i.e. local, state, national and international)	Maintenance of existing partnerships with universities and health services Formulation of memorandums of understanding with universities and other organisations (including health services and industry partners) Hosting and development of annual regional occupational therapy in mental health research symposium
	Demonstrate and document on-going development of research and knowledge translation culture	Development and annual review of Occupational Therapy Research Strategy, and associated Annual Report

Note: *AUD* = Australian Dollars

## Discussion

This study has provided a multi method evaluation of the impact of a leadership position for research and knowledge translation in occupational therapy. This position has been successful in influencing research culture, and achieving a tangible impact across areas identified in previous literature about similar positions in allied health.

Academic outcomes (such as participation in formal quality assurance and research proejcts, publications, conference presentations, grants and higher degrees by research student supervision) are the most easily quantified. Such outcomes increase the sustainability of such positions in several ways, by supporting the career progression of incumbent academics, founding a track record to attract funding, providing opportunities for clinician professional development, and disseminating research findings and knowledge translation for the broader professional good. Perry et al. [18] noted that records of such outcomes are not always kept in health services, potentially due to the perception they are only relevant in academic circles. The Lead Research Occupational Therapist has spent significant time highlighting the meaning of these outcomes to clinical work, as these links were not initially recognised by the workforce. A database of knowledge translation activities is now in place in this service, and referred to regularly.

It is difficult to benchmark the number of publications and presentations reported in this study, as there are few comparisons available. Around one third of the 24 bursary and grant recipients in the study by Ried [17] published their work, while approximately one half had presented at conferences. The capacity building approach in this study has included a focus on publication skills, which are recognised as a potential barrier to clinicians disseminating research [14]. Some similar positions have also included elements of student supervision [12, 37], which have in this service also contributed to publication and conference presentations for the service.

The research performance outcomes (such as participation inquality assurance and knowledge translation activities, and mentorship) are also relatively visible. Previous studies in allied health have found that the highest qualification attained was a predictor of research generation [38], but all of the Masters qualified occupational therapists in this sample undertook those degrees as pre-registration qualifications (rather than as research training). The smaller increase in clinicians identifying themselves as research leaders may be related to the relatively fewer recognised leadership positions available in the profession [39]. While clinicians can attain research leadership at any stage of their career, via development of specialist skills and knowledge, they may still associate it with seniority in rank.

The presence of projects at all stages of the research process (from proposal formation to publication) demonstrates how the position described here supports individual clinicians and/or teams through a projects lifespan. Similar findings have also been reported in other studies of similar roles, where incumbents are required to manage multiple projects at various stages of development [12, 16–18, 37, 40]. Individual mentorship has also been consistently cited as an effective intervention for improving research participation for allied health clinicians [41–43], and the position described here overtly utilises these proven strategies to support research and knowledge translation.

However, a significant point of difference for the position reported here has been its focus on developing a culture of knowledge translation, rather than research or evidence based practice. The terms ‘research’ and ‘evidence based practice’ continue to have a presence due to clinicians greater familiarity with them, however ‘knowledge translation’ is beginning to become more prevalent in discussions at a service level. The continuing positive attitudes of this workforce towards evidence-based practice reported here are solid foundations on the emergent knowledge translation culture has been built. While attitudes do not always translate into behavioural changes, the motivation and persistence required to engage with the complex task of knowledge translation cannot be expected in their absence.

The social analysis network utilised in this study provided confirmation of changes anecdotally reported by the workforce, and clearly demonstrates how this method can visualise relationships which either facilitate or impede knowledge translation. The decrease in ‘bottlenecks’ [44], where a single person from a service is the only connection to other services, has enabled greater dissemination across this large service, including the roll out of several interventions across multiple programs. The increase in the number of contacts between clinicians indicate that evidence based practice and knowledge translation are now part of the regular discourse in the service. Denser networks have previously been found to promote the greater dissemination of information and knowledge [45]. Overall, the metrics provide support for an increased awareness of human knowledge capital within the service, greater access to that knowledge and increased engagement, coordination and interaction. These findings are important because they measure changes in behaviour (rather then just attitudes or self reported knowledge), and illuminate the impact of the position on the communications and culture of the organisation [32, 44]. The significant increase in preferring online contact (rather than face to face) may be due to the large geographical area covered by this service, but the use of this mode of communication as a primary means of contact around evidence based practice could be worth exploring in future.

The workforce in this study cited multiple examples of knowledge translation into their practice, as have clinicians in previous research [46]. Given the importance of local context to knowledge translation, consistently measuring impact on practice can be challenging [47]. The collection of specific examples in case studies could support the task of adaptation, illustrating measures that succeeded across a range of settings and circumstances. Partnerships with universities are often the foundation of positions such as these [15], although additional partnerships with neighbouring health services and industry partners have also been key contributors to the outcomes reported. The Lead Research Occupational Therapist has also pursued active participation in governance structures and other interdisciplinary networks as a means of supporting knowledge translation. Leadership support, communication, physical and financial resources are known to be crucial mechanisms for knowledge translation [46], however, the role of governance structures remains largely unexplored at this time. Allied to these structures are opportunities for non-academic documentation (such as clinical documentation, research reports and electronic communication), as a means of embedding knowledge translation and increasing its visibility across and beyond the service.

While this position has achieved many tangible outcomes so far, this evaluation has highlighted an important area of neglect. Client centredness is a core value of occupational therapy, and co-production of knowledge translation initiatives with consumers and carers is beneficial to both consumers and health professional [48]. However, the findings indicated the workforce are not sharing quality assurance and research outcomes with patients and/or carers (or completing the knowledge translation process by evaluating the impact of new practices). The challenges of co-production include logistical issues, reimbursement and time to establish the necessary relationships [49, 50]. The service in this study has partially overcome these by consulting the consumer advisory group for some projects and co-authoring a publication with two consumers. However, a more systemic approach is required and changes are currently being made to achieve this.

The generally positive perceptions of the Lead Research Occupational Therapist role across a range of characteristics is congruent with clinician perceptions of similar roles [14, 46]. The identification of discipline specificity as the most valued characteristic reflects informal feedback from the workforce, who report the occupational therapy focus is key to achieving valued outcomes. A deep knowledge of the disciplinary, clinical and organisational environment in which clinicians operate, is likely to be advantageous to guiding knowledge translation, beyond the research skills and knowledge that are also bought to the role.

### Limitations

There are several limitations to acknowledge in this study, the foremost of which is its containment within a single service. The size of this service supports an unusually large and diverse occupational therapy workforce, and its size undoubtedly contributed to the service’s ability to create and maintain the Lead Research Occupational Therapy role. Context is recognised as a crucial factor in knowledge translation, and this will limit the ability to generalise the findings reported here more broadly. Around 50% of the available workforce responded to these surveys, however a more representative sample would have provided further information about the social network, which could have been important to understanding its structure. The samples were different across time (with staff attrition influencing the membership of the workforce), and this may also have influenced the findings, as the design did not provide for matched pre-post sample and measures. Tracking outcomes over time for clinicians in a matched sample would enable the exploration of the potential impact of these roles at the individual level. The workforce in this service is very interested and keen in research and knowledge translation, and this may not be true of other workforces in other services. Finally, the analysis here does not take into account the specific activities and proportional time spent by the Lead Research Occupational Therapist to achieve these outcomes. A greater understanding of this ‘active ingredient’, described by some as the ‘black box’ of implementation science [51], is required to fully understand the longer term sustainability and viability of this position.

## Conclusions

The findings of this study have demonstrated that a leadership position focussing on knowledge translation in occupational therapy made a tangible impact in its initial years. It succeeded in meeting all of the key performance indicators set at its inception, across the academic, research production and cultural domains. As a result, the capacity of the workforce to engage in knowledge translation has improved, and practice has changed in response to the best available evidence.

This study has made a significant contribution to the literature around similar positions in allied health, by addressing gaps in current knowledge. In particular, social network analysis was found to be an effective tool for understanding the interpersonal relationships that are so crucial to knowledge translation, and should be considered as part of future evaluations of similar positions. The sustainability of partnerships such as the one described here depends on evidence supporting their effectiveness, and this study supports the effectiveness of taking a knowledge translation focus.

## Abbreviations

EBPAS-15: Evidence Based Practice Attitudes Scale 15
